# Perioperative micro-arterial function and extravasation in cytoreductive ovarian cancer surgery: an observational study

**DOI:** 10.1186/s40635-025-00839-4

**Published:** 2026-01-25

**Authors:** Aarne Feldheiser, Jana-Jennifer Dathe, Sandra Heinig, Klaus Pietzner, Lutz Kaufner, Oliver Hunsicker, Clarissa von Haefen, Jalid Sehouli, Claudia Spies

**Affiliations:** 1 Department of Anesthesiology and Operative Intensive Care Medicine (CCM, CVK), Charité - Universitätsmedizin Berlin, corporate member of Freie Universität Berlin, Humboldt-Universität zu Berlin, and Berlin Institute of Health, Berlin, Germany; 2 Department of Gynecology with Center for Oncological Surgery, Charité - Universitätsmedizin Berlin, Campus Virchow Klinikum, Berlin, Germany; 3 Department of Anesthesiology, Intensive Care Medicine, and Pain Therapy, University Hospital Knappschaftskrankenhaus Bochum, Ruhr-Universität Bochum, Bochum, Germany

## Abstract

**Background:**

Patients undergoing extended multivisceral, non-cardiac surgery require a high demand for intravenous fluid administration, leading to substantial positive fluid balances. This study aimed to perioperatively characterize extravasation as a correlate of capillary leakage and micro-arterial regulation, as well as venous return characteristics, as possible causes for the positive fluid balances.

**Methods:**

In this single center, observational trial we included patients undergoing abdominal debulking surgery due to ovarian cancer. The measurements were performed by a venous congestion plethysmography (VCP) protocol to determine extravasation, micro-arterial reagibility, and after deflation of congestion venous outflow characteristics at timepoints before and during surgery, and repeatedly during the postoperative course.

**Results:**

Thirty patients with primary ovarian cancer undergoing cytoreductive surgery treated within a goal-directed hemodynamic algorithm (GDA) based on the esophageal Doppler were included in the analysis. Stroke volume index did not change throughout the procedure with an increase in heart rate and consequently an increase in cardiac index. The norepinephrine requirements to maintain mean arterial pressure increased during surgery. Patients received a median 1750[25-quartile 1075;75-quartile 2100]ml crystalloids and 1000[1000;1500]ml starches, transfusions of 0[0;1040]ml red-packed cells, and 360[0;2880]ml fresh-frozen plasma. The intraoperative fluid and blood loss of 1020[508;1695]ml resulted in a positive fluid balance (2820[1338;6075]ml). Extravasation did not increase during surgery, even in the presence of substantially positive fluid balances. On the third and fifth postoperative days, extravasation increased relative to the preoperative baseline value. The micro-arterial function deteriorated throughout the course of the surgery, recovering to baseline values within 4 h after surgery. The venous outflow characteristics of the limb after releasing the venous congestion deteriorated over the course of surgery.

**Conclusions:**

There was no increase in extravasation measured by VCP during surgery despite a substantial intraoperative positive fluid balance, showing that they were not associated with each other. The micro-arterial function and venous backflow characteristics deteriorated during surgery, indicating that vascular dilation rather than capillary leakage may contribute to the high fluid demands.

*Trial registration*: ClinicalTrials.gov identifier: NCT01311297.

**Supplementary Information:**

The online version contains supplementary material available at 10.1186/s40635-025-00839-4.

## Introduction

Fluid administration to preserve preload is considered a cornerstone of hemodynamic therapy perioperatively [1]. However, excessive fluid administration leading to perioperative weight gain is associated with a risk of postoperative complications, such as sepsis, pulmonary edema, or myocardial arrhythmias [2]. Moreover, the intravenous fluid volume on the day of surgery was the strongest predictor of postoperative complication [3]. Nevertheless, data examining intravascular hypovolemia leading to a decrease in stroke volume are associated with the risk of splanchnic hypoperfusion [4]. Goal-directed studies have shown that it is not only the amount of fluid but also the timing of fluid administration, with initial fluid administration being superior to fluid administration at the end of surgery [5]. However, even fluid administration using a goal-directed hemodynamic treatment algorithm that optimizes not only fluid administration but also vasopressor administration was associated with a substantial positive fluid balance independently of the choice of intravenous solution [6]. Possible reasons for a positive fluid balance in a patient undergoing surgery might be increased extravasation—often referred to as capillary leakage—or reduced lymphatic drainage leading to increased tissue edema. Alternatively, elevated venous pooling due to vascular dilation in combination with parallel fluid administration could lead to an increased circulatory blood volume as a possible explanation for the positive fluid balance.

It has been demonstrated in humans [7–9] that inflammation, sepsis, trauma, and hemorrhage all lead to disruption of the endothelial surface layer (ESL). In clinical settings, when the ESL is disrupted, vascular fluid leakage may lead to capillary leak syndrome and promote edema formation, with all their consequences [10]. In addition, ESL disruption may lead to intravascular hypovolemia associated with organ hypoperfusion [11]. However, in humans, evidence of perioperative ESL shedding associated with clinically relevant vascular fluid leakage is based on increased laboratory values. The only study demonstrating vascular fluid leakage during surgery via direct functional measurement was more than 25 years ago [12]. The protocol for the measurement took more than 30 min, and it was only demonstrated in vascular surgery. In the meantime, perioperative care has substantially changed, and the relevance of these data remains unknown. In addition, direct differences in volume kinetics observed in multiple studies reviewed by Hahn et al. undermine our sparse understanding of the effects of fluid therapy and consequently the interaction to vascular function [13, 14]. This underscores the need to gather clinically relevant functional data on perioperative vascular leakage.

The micro-arterial regulation assesses the capacity of arteries and arterioles to constrict or dilate due to arterial blood flow changes. It is referred to as flow-mediated vasodilation mediated by the vascular endothelium liberating nitric oxide [15]. Decreased micro-arterial function is associated with increased mortality in patients after myocardial infarction [16]. Micro-arterial function was previously shown to deteriorate throughout the course of surgery [17]. Furthermore, it was highly associated with norepinephrine requirements in gynecological debulking surgery [18]. However, the time of functional recovery remains to be determined in the postoperative period.

In this study, we hypothesized that extravasation deteriorates throughout surgery and that it is highly associated with a positive fluid balance even within a goal-directed hemodynamic algorithm to guide fluid and catecholamine administration. Therefore, we aimed to measure extravasation perioperatively using mercury in silastic strain-gauge venous congestion plethysmography (VCP) as a direct, functional methodology. In addition, this methodology assesses micro-arterial reagibility and venous outflow patterns following venous congestion. To our knowledge, this is the first direct measurement of extravasation and venous outflow using a direct methodology in recent years.

## Materials and methods

### Study design and participants

This was a prospective, observational, monocentric study conducted at the Campus Virchow Klinikum of the Charité—Universitätsmedizin Berlin, Germany. Ethical approval for this study was provided by the responsible ethics committee of the Charité—Universitätsmedizin Berlin, Germany (Ethics committee N° EA1/004/11, Chairman Prof. Dr. med R. Uebelhack). This study was registered internationally (ClinicalTrials.gov ID: NCT01311297, 2011) prior to patient recruitment that was performed from March 2011 to September 2012. Eligible patients were adults who underwent debulking surgery due to primary ovarian cancer or ovarian cancer recurrence and provided informed written consent to participate in the study. In the study project, it was planned to include 30 perioperative patients, 30 obstetric patients, and 15 healthy controls. The study recruitment plan was fulfilled in the perioperative arm and for the healthy controls. However, this analysis was limited to patients who underwent surgery (see consort flow diagram in the supplement diagram 1).

Further criteria for exclusion were age minor to 18 years, an ASA Score ≥ 4, chronic heart failure NYHA IV, radiological evidence of pulmonary edema, dialysis-dependent kidney insufficiency, atrial fibrillation, severe illness of the esophagus or the upper airways (within the last 2 months), venous thrombosis (within the last 3 years), intracranial hemorrhage (within the last year), diabetic neuropathy, intolerance to colloidal fluids, unclear alcohol use history, neurological or psychiatric disorders limiting their legal capacity, being institutionalized due to official order, or being an employee of the Charité.

The patients were evaluated, prepared for surgery, and treated according to the published and certified standards (according to DIN EN ISO 9001) of the Department of Anesthesiology and Intensive Care Medicine, Campus Virchow-Klinikum and Campus Charité Mitte, and of the Department of Gynecology with Center for Oncological Surgery, Campus Virchow-Klinikum, Universitätsmedizin Berlin, Germany. Throughout the hospital stay, no diagnostic or treatment decisions were made based on the data. Clinical data (preoperative patient characteristics, intraoperative hemodynamic data, pre- and postoperative laboratory values, and postoperative clinical data) were prospectively collected and analyzed.

To standardize and optimize hemodynamic monitoring and care, we treated the patients within a goal-directed hemodynamic algorithm, as published previously [19]. Briefly, it guides fluid administration based on the stroke volume measured by esophageal Doppler and maintains mean arterial perfusion pressure by continuous administration of a vasopressor, and if the patient is still in a low-cardiac output state, positive inotropes are indicated. An international group of experts on enhanced-recovery-after-surgery pathways developed the algorithm and tested and validated it internationally [19].

### Experimental setting

Vascular characteristics were assessed using mercury in silastic strain-gauge venous congestion plethysmography (VCP) (Vasolab 5000, ELCAT GmbH, Wolfratshausen, Germany) preoperatively (day prior to surgery), hourly throughout the surgery, postoperatively 1 and 6 h after surgery as well as on days 1, 3, and 5 after surgery. Before the experimental study visits, the day before and the days after surgery, all patients refrained from physical activity for at least 30 min and were asked to remain during the measurements. Pneumatic cuffs with multiple air inlets to permit rapid inflation were applied to the right upper arm approximately 3–5 cm proximal to the cubital joint. The junction head of the strain-gauge sensor was attached to the forearm at the largest circumference of the forearm using double-stick discs. Hereafter, strain-gauge sensors of appropriate length were applied ensuring a tight-fitting placement on the skin and minimum pretension. Volume change (VC) was measured by a changed stretch of the sensors. The arm was abducted 45° in conscious patients, and as required throughout surgery applying pads distal from the strain gage mountain, ensuring free positioning of the strain gage sensor.

### Experimental protocol

As described previously [20], members of the study group performed all measurements that were well-trained by the company providing the device and by a series of test patients to assure reproducibility. Data acquisition and processing were fully automated. VCP was started without pressure, during which resting data were assessed. Resting data were recorded when two serial measures did not differ by more than 4 s. Thereafter, a single cuff pressure increase was performed to stop venous outflow (60 mmHg) and was maintained at this level throughout the examination. Assessing the steepness of the initial volume change (VC) determined the micro-arterial characteristics of the forearm that reflected micro-arterial dilation. During the first 3 min, limb volume increases exponentially due to the rapid volume response (RVR), which initially describes the change in blood vessel volume due to compliance of the vessels and surrounding tissues. If there were implausible values of the baseline or the starting curves, the measurement was stopped. This almost always occurred due to movement of the arm. In those cases, the measurement was restarted after correcting the position.

**Fig. 1:**
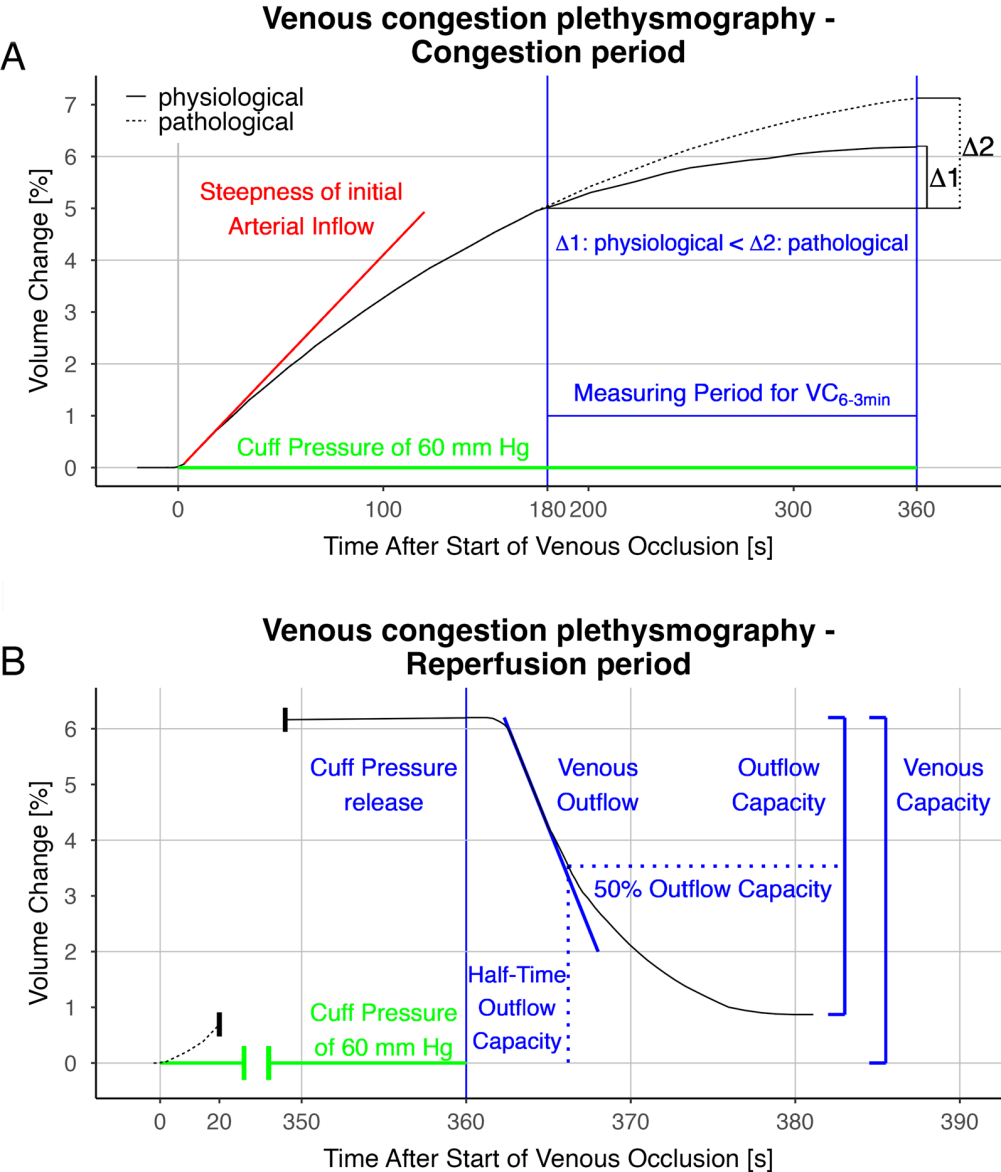
Schematic figure to visualize the parameter of the venous congestion plethysmography during congestion and reperfusion. **A** Congestion period enables the measurement of arterial inflow as a marker of micro-arterial function and the volume change between the third and the sixth minute (VC _6–3 min_) as a marker describing extravasation. **B** During the reperfusion period, the velocity of venous outflow is characterized by the Venous Outflow with the steepest downslope and the half-time of the outflow capacity. Venous capacity is characterized by the outflow capacity and the venous capacity

Fluid filtration was assessed by the difference in the volume changes (VC) of the limb between the third and sixth minutes during venous congestion (VC_6–3 min_) (Fig. 1A). The linear increase in limb volume was due to extravasation of fluids and blood entering the limb to replenish the extravasated volume. According to data from the literature and our previous publication [20–22], our experimental protocol assessed fluid filtration after 3 min (180 s), ensuring that the RVR was not relevant anymore. Implausible values for VC6–3 min (negative values or values > 5%) were excluded from the final analysis.

With the deflation of the cuff pressure, the venous blood could outflow into the congested limb. The negative volume change in the limb over the following 30 s characterized the venous outflow pattern. The measurement protocol was completed (Fig. 1B).

### Measurements of interleukin 6 (IL-6) and intercellular adhesion molecule 1 (ICAM-1)

Whole blood was collected from patients in EDTA tubes, and plasma was obtained by centrifuging the tubes at 2500 × g for 10 min and then stored at − 80 °C until analysis. IL-6 and ICAM-1 levels in plasma were quantified using the Human Interleukin 6 (IL-6) ELISA Kit (catalog number: RD-IL6-Hu) and the Human Intercellular Adhesion Molecule 1 (ICAM-1) ELISA Kit (catalog number: RD-ICAM1-Hu) from Reddot Biotech Inc. (Kelowna, British Columbia, Canada) according to the manufacturer’s instructions. The concentrations of IL-6 and ICAM-1 were calculated using a standard curve. Measurements were performed preoperatively, postoperatively 1 and 6 h after surgery as well as on days 1 and 3 after surgery.

### Statistical analysis

This is an exploratory pilot study. No sufficiently reliable data about differences between the defined clinical parameters were available for planning the study. For this reason, no statistical calculation of the sample size could be accomplished.

Data processing and analysis were performed using the programming language R for statistical computing (Version 4.1.1 [23]; R-packages used were Gmisc, Hmisc, nparLD [24], coin, tableone, htmlTable, tidyverse, knitr, rmarkdown, rstatix, scales, gridExtra), and the software R Studio^®^ (Version 1.4.1717) [25]. Differences between matched patient data were evaluated using the Wilcoxon signed-rank test for metric data and Fisher’s exact test for categorical variables. A non-parametric longitudinal data analysis in a one-factorial experiment was used to assess changes in VCP and clinical data over time (ANOVA, dependent factor time) [24]. This test was used for direct and relative comparisons of VCP parameters with preoperative baseline values and for IL-6 and ICAM-1 analysis. A two-tailed *p* value of 0.05 was considered statistically significant. Nevertheless, due to the study design, all *p* values feature an exploratory character and thus do not allow for generalization or proof. For the same reason, no alpha adjustment for multiple tests was conducted. The data are expressed as median [quartiles] or frequency (%) according to the scale.

## Results

Thirty patients were included in the study. 17 patients (15 control patients and 2 pregnant patients) did not fulfill the criteria for this perioperative analysis (see supplemental diagram 1). The data showed that all patients had plausible data, indicating that the methodology of the VCP was feasible in the perioperative setting.

### Patient and oncological characteristics

Preoperative characteristics showed that patients had a median Charlson comorbidity index of 6 [25% quartile: 6; 75% quartile: 6], mainly due to the cancer diagnosis without a relevant incidence of cardiovascular comorbidities. Gynecological–oncological relevant was the presence of ascites in 7 out of 29 patients (23.3%) as an indicator of advanced disease (see supplemental Table 1).

### Intraoperative and postoperative hemodynamic values and fluid administration

During surgery, hemodynamic data were obtained within a goal-directed hemodynamic algorithm based on the esophageal Doppler and showed a stable stroke volume index and mean arterial pressure, whereas the heart rate increased continuously throughout the surgery, leading to an increased cardiac index. The corrected flow time as an indicator of afterload did not change during surgery, whereas the required norepinephrine administration increased continuously for an unchanged mean arterial pressure (see supplemental Fig. 1 and supplemental Table 1).

**Fig. 2:**
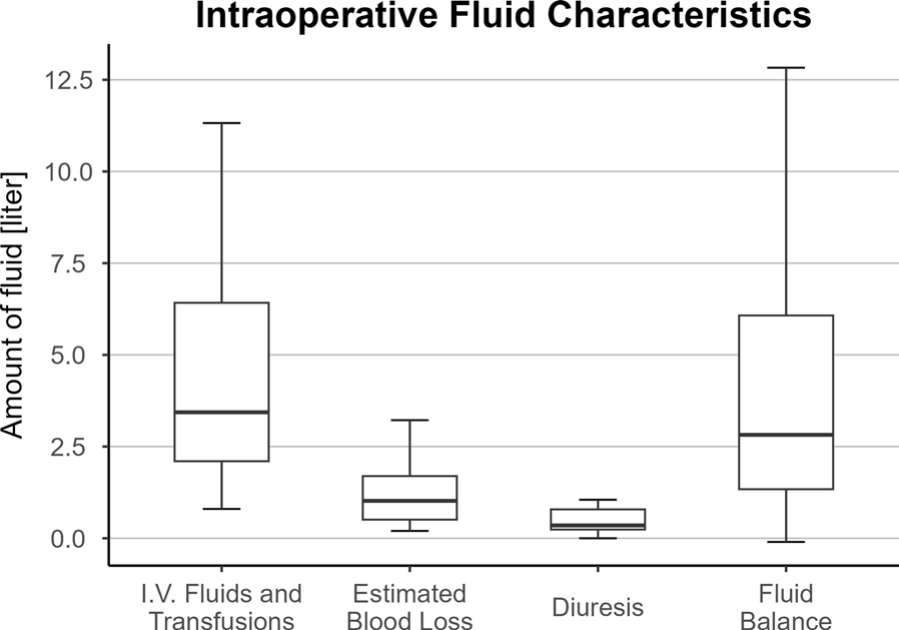
Intraoperative fluid characteristics

The patients received 1750 (1075; 2100) ml of crystalloid, 1000 (1000; 1500) ml of colloid solutions, 360 (0; 2880) ml of fresh-frozen plasma, and 0 (0; 1040) ml of red-packed cells during surgery. With 1020 (508; 1695) ml of intraoperative fluid loss, the resulting intraoperative fluid balance was substantially positive with 2820 (1338; 6075) ml (Fig. 2).

**Fig. 3:**
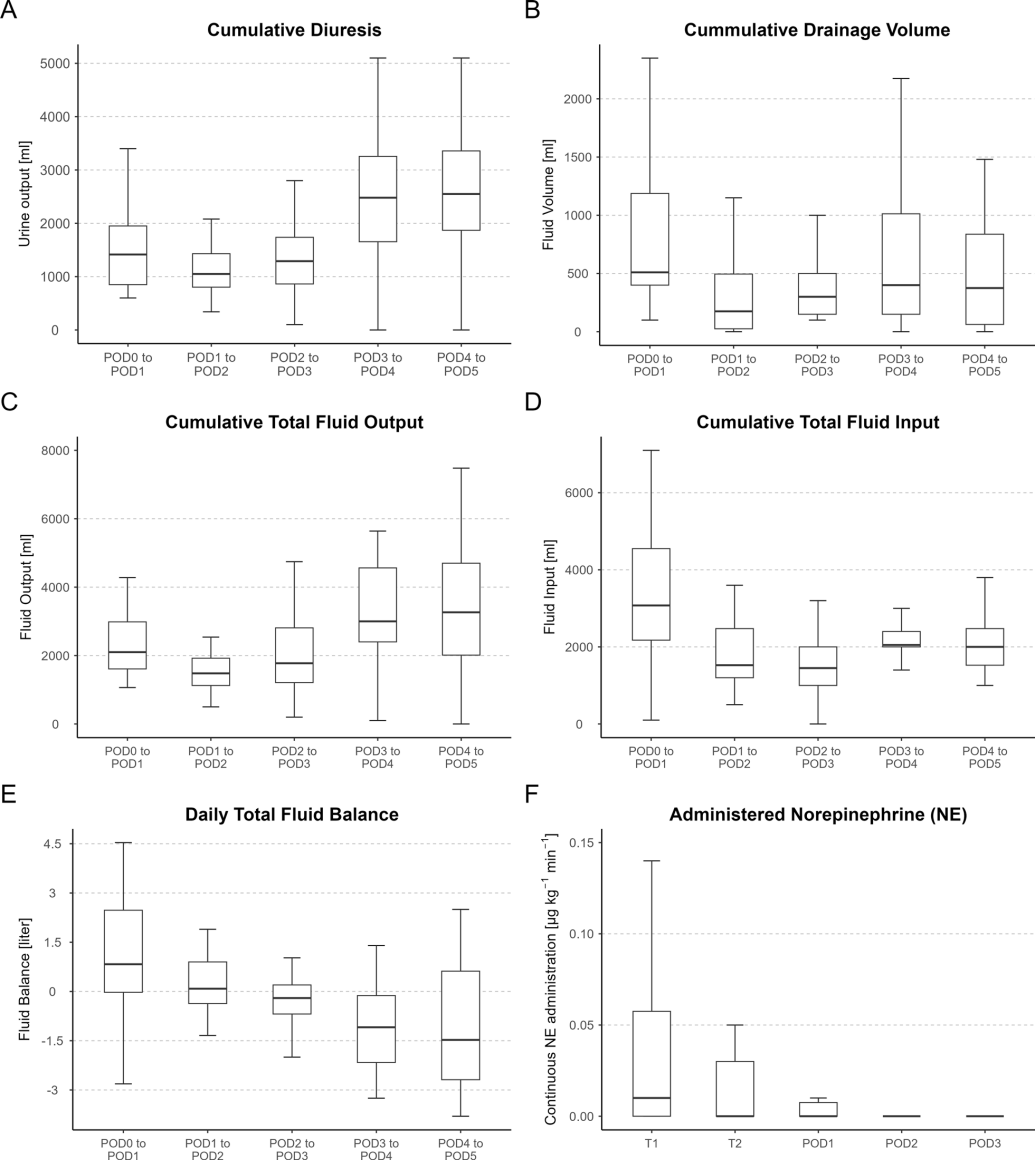
Postoperative time course of fluid parameter and norepinephrine administration. The values represent cumulative values of the outlined time periods. The figure of norepinephrine administration shows the values at the outlined timepoints

Postoperatively, descriptive data are shown in Fig. 3 with the time course of postoperative diuresis, drainage volume, total fluid output, and input summarizing the time course of total fluid balance. Postoperative norepinephrine requirements showed a steady decline in administered doses, with requirements from the second postoperative day on being the exception (Fig. 3).

### Characteristics of extravasation, micro-arterial function, and venous outflow

**Fig. 4:**
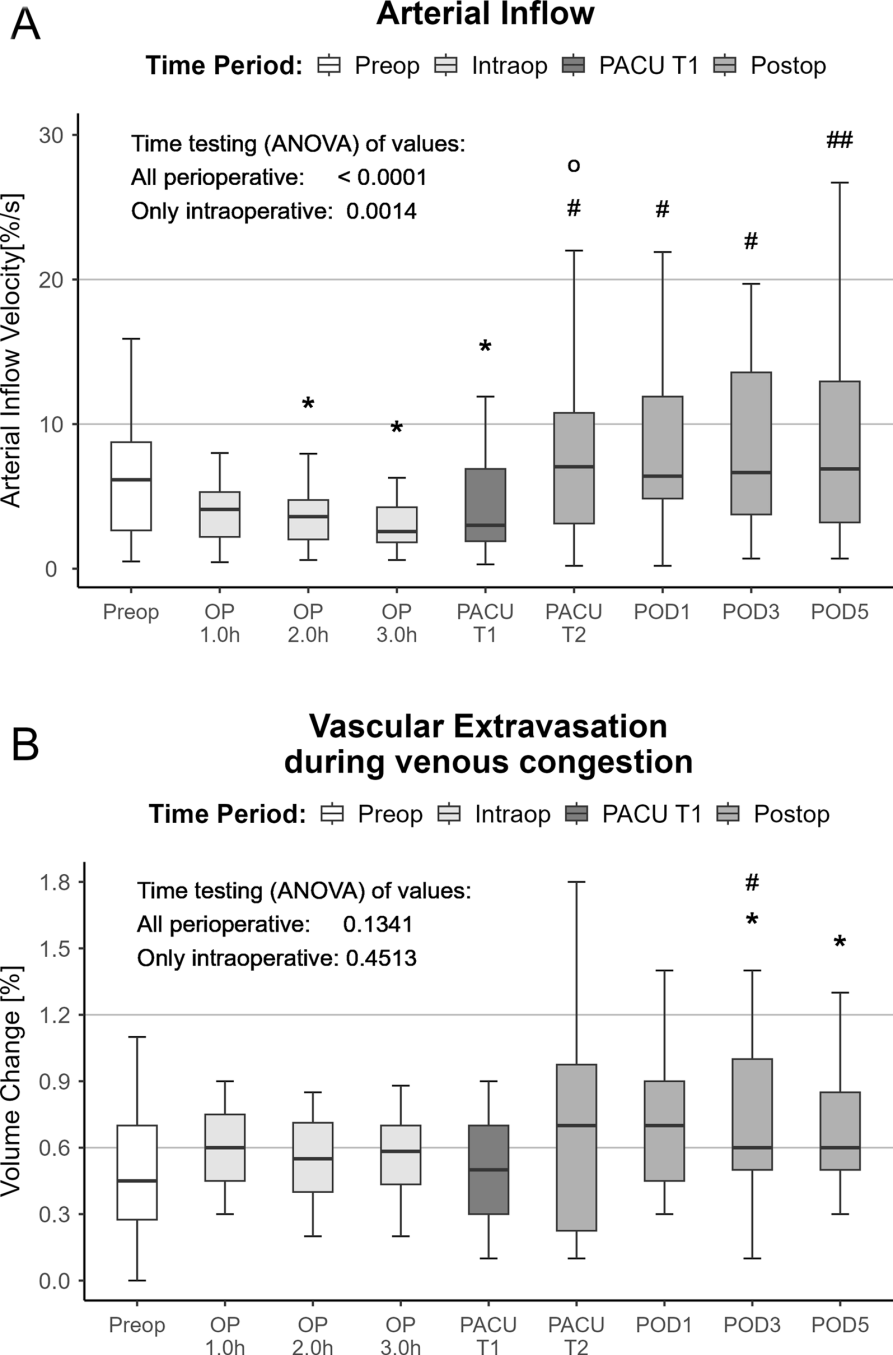
Perioperative time course of the micro-arterial function and the extravasation. Time (ANOVA) effects were tested over all perioperative values (all perioperative) and for the intraoperative time course (preop to OP 3.0 h; only intraoperative). Asterisks indicate (*) *p* < 0.05 versus preop values. Sharps represent (#) *p* < 0.05 and (##) *p* < 0.01 versus the last intraoperative values (OP 3.0 h). Circles indicate (o) *p* < 0.05 versus first postoperative values (PACU T1)

The steepness of the arterial inflow initially after the cuff pressure increase characterized the micro-arterial function and showed a decrease from preoperative values [2.7 (0.5; 6.2)%/s] to the measurements during surgery [1.8 (0.6; 2.6)%/s]. Directly after surgery, arterial inflow was still decreased but recovered already 4 h after surgery [3.1 (0.2; 7.1)%/s] compared with baseline values (Fig. 4A).

**Fig. 5:**
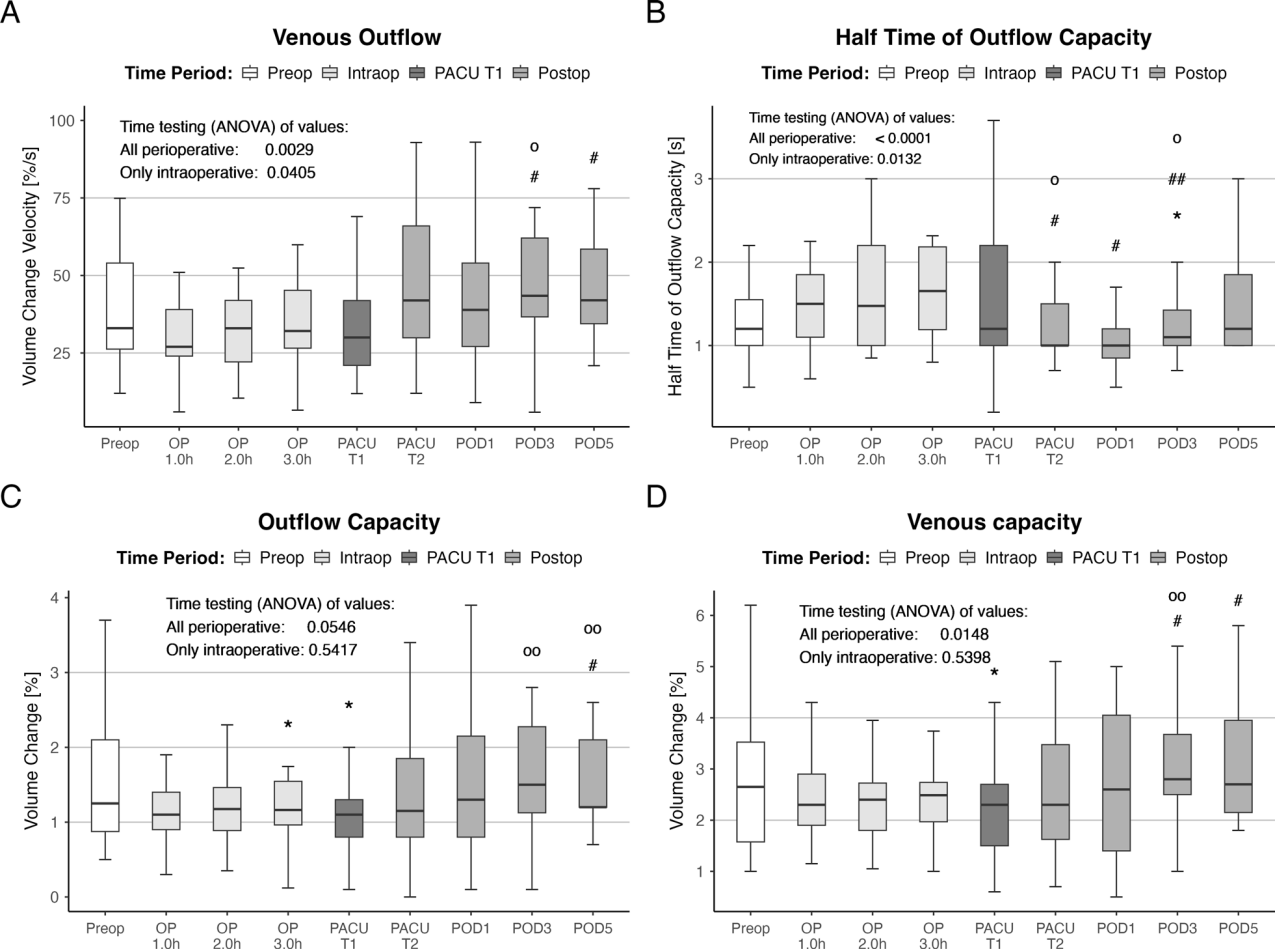
Perioperative time course of venous outflow (venous outflow and half-time of outflow capacity) and venous capacity parameter (outflow and venous capacity). Time (ANOVA) effects were tested over all perioperative values (preop to POD5; all perioperative) and for the intraoperative time course (preop to OP 3.0 h; only intraoperative). Asterisks indicate *p* < 0.05 versus preop values. Sharps represent #*p* < 0.05 and ##*p* > 0.01 versus the last intraoperative values (OP 3.0 h). Circles indicate o *p* < 0.05 and oo *p* < 0.01 versus first postoperative values (PACU T1)

The measured values for extravasation during venous congestion were unchanged compared with the preoperative values during surgery. During the third and fifth postoperative days, extravasation increased significantly (Fig. 4B). The parameter characterizing the venous outflow pattern after the release of the positive pressure cuff indicated that the initial phase showed a decreased velocity of volume change. This was accompanied by an unchanged outflow capacity. During the postoperative course, the velocity of volume change increased significantly compared with the last intraoperative value, and the outflow capacity increased similarly compared with the intraoperative value (Fig. 5).

### Perioperative laboratory findings

**Fig. 6:**
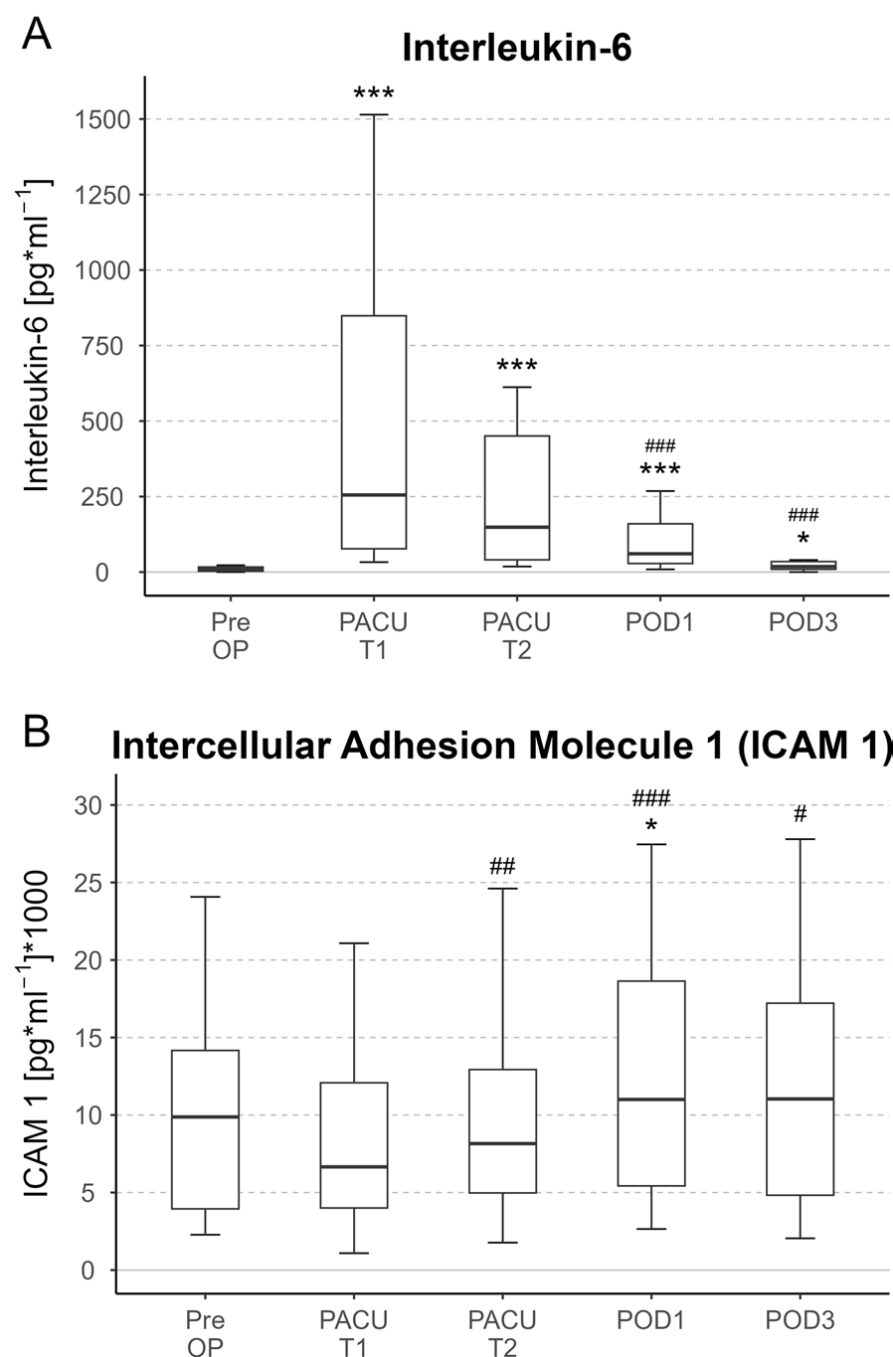
Perioperative time course of interleukin-6 and intercellular adhesion molecule 1. Asterisks indicate * = *p* < 0.05 and ***p < 0.001 versus preop values. Sharps represent ##*p* < 0.01 and ###*p* < 0.001 versus the first postoperative values (PACU T1)

Perioperatively, IL-6 levels initially increased 1 h after surgery and continuously declined but did not normalize to preoperative levels until the third postoperative day (Fig. 6). ICAM-1 levels increased compared with baseline values only in the morning after surgery and compared with the ICAM-1 levels immediately after surgery already 6 h after surgery, up to the third postoperative day. The plasma concentrations significantly changed during the postoperative course (Fig. 6). Protein and albumin plasma levels decreased substantially at the end of surgery and recovered up to the fifth postoperative day. Creatinine levels spiked in the morning after surgery but recovered instantly the next postoperative day (supplement_Figure_2). In contrast, bilirubin levels were directly elevated after surgery and recovered up to the second postoperative day. nt-pro-BNP levels spiked the morning after surgery and did not normalize up to the fifth postoperative day (supplement_Figure_2). C-reactive protein levels were directly decreased after surgery compared with preoperative values and increased from the morning after surgery and remained elevated up to the fourth postoperative day (supplement_Figure_2).

## Discussion

In this study, we examined micro-arterial function and extravasation characteristics as well as venous outflow patterns in patients undergoing laparotomy for debulking due to ovarian cancer. Related to the complex, multivisceral extent of the surgery, moderate volumes of intravenous fluids and transfusion of blood products were administered using a goal-directed hemodynamic algorithm. Nevertheless, this resulted in a substantially positive intraoperative fluid balance. In contrast, the analysis of extravasation showed no increase compared with preoperative values throughout surgery. These results indicate that extravasation was not a major factor contributing to substantially positive intraoperative fluid balance. However, on the third and fifth postoperative days, we measured increased extravasation compared with preoperative and intraoperative values. The micro-arterial function deteriorated throughout surgery but quickly recovered after surgery.

The intraoperative time courses of hemodynamic values such as stroke volume, cardiac index, heart rate, and norepinephrine requirement were comparable to those of previous hemodynamic studies in patients undergoing multivisceral debulking due to ovarian cancer [6, 26]. This showed that the goal-directed algorithm based on the esophageal Doppler could stabilize intraoperative hemodynamics by guiding intravenous fluid and catecholamine administration. However, the use of goal-directed fluid administration did not prevent a profoundly positive fluid balance. This fact is consistent with data previously reported in our own studies [6, 26] and in the literature on this complex surgical procedure [27].

The relevance of extravasation and the association to peripheral edema was shown previously in the perioperative context [20]. Interestingly, extravasation measurements performed on the arm showed a higher association based on a grey-zone analysis with the clinical features of peripheral edema than the measurements on the legs. Therefore, we consider the methodology for perioperative patients suitable for determining fluid filtration via venous congestion plethysmography. The intraoperative measurements were feasible, because we could position the left arm sufficiently well for the measurements. Moreover, with a protocol of slightly more than six minutes, it was short and did not impair clinical work.

In the publication assessing the methodology, a grey-zone approach to associate the values of extravasation to the presence of edema found an upper limit of the small grey zone of 0.7% volume change for the measurements on the forearm. As metastatic ovarian cancer must be considered as systemic disease, it is important to note that preoperatively only one outlier had higher values than 0.7% volume change the day before surgery. This indicates that despite the metastatic disease at baseline, no increased extravasation was shown in the study population. In contrast to the positive fluid balance observed during surgery, the data on extravasation showed no change compared with preoperative values throughout the course of surgery. These results carefully indicate that there might not be an increase in extravasation during surgery that contributed to the positive fluid balance. However, the extravasation deteriorated during the postoperative course and increased compared with preoperative and intraoperative values. Interestingly, at that timepoint, the total fluid balance was already turning negative, and the patients were hemodynamically stable without the requirement for norepinephrine. According to our knowledge, clinical data describing extravasation were not available during the recent years. Data published in 1999, based on a prolonged protocol of 30 min per measurement cycle, indicated intraoperative increased extravasation during vascular surgery [12]. The question remains whether these results are transferable to abdominal surgery. In addition, during the last 25 years, perioperative care has made substantial progress, further explaining the different results. Perioperative fluid management was shifted to shorter fasting times and immediately starting fluid intake after surgery. It is possible that liberal fluid administration during anesthesia induction [2] contributed to extravasation in the earlier studies [11]. Furthermore, in this study, a goal-directed hemodynamic algorithm based on the esophageal Doppler was used to optimize intraoperative fluid management, as previously reported [19].

After releasing the venous congestion for six minutes, venous outflow indicated an impact on the venous characteristics during the perioperative course. In our data, the velocity of volume change and the half-time of outflow capacity characterized the venous outflow, and both parameters indicated a slower backflow of venous blood to the heart. In the immediate postoperative period, the outflow capacity and venous capacity decreased compared with baseline values, indicating a decreased ability of the veins to dilate during the stop-flow period. According to our knowledge, this is the first study to perioperatively describe venous function by direct VCP measurements of volume changes and venous reagibility. These data point toward the venous function as a relevant research topic for upcoming studies.

Dysfunction of the micro-arterial regulation to constrict or dilate arteries and arterioles according to blood flow changes predicts increased mortality in patients after myocardial infarction [15] and is in the general population a risk factor of death [16]. Our analysis of the micro-arterial function induced by rapid cuff inflation revealed a decrease in the micro-arterial reacting capacity throughout the course of the surgery. This is consistent with our previous data of near-infrared spectroscopy combined with a vascular occlusion test in the same patient population, which were equally optimized within a goal-directed hemodynamic algorithm [18].Perioperative data of other groups in the literature [17] showed the reliability of our data. In our previous study, we have shown that the micro-arterial function is associated with amongst other to the norepinephrine requirements to maintain mean arterial pressure in patients undergoing debulking surgery. Taken together with the long-term findings in the field of cardiology of the micro-arterial dysfunction [15, 16], we think that the intraoperative decline is clinically relevant and merits further research in the future. The novel approach in this study is the measurement of micro-arterial function throughout the entire perioperative period. Interestingly, the data showed rapid postoperative recovery after surgery and normalized versus preoperative values already at the timepoint 4 h after surgery. At this timepoint, extravasation deteriorates during the postoperative period, and the micro-arterial reacting capacity is not affected. This undermines the notion that micro-arterial function and extravasation are not associated with each other; but rather must be considered as independent pathophysiological features in perioperative care. Our previous analysis [18] showed a high association between impacted micro-arterial function and norepinephrine requirements during the intraoperative period. In this study, the norepinephrine requirement was weaned within a short period after surgery being highly associated with the postoperative recovery of the micro-arterial function during the postoperative period. Interestingly, the corrected flow time (FTc), which characterizes afterload, remained unchanged throughout surgery. This result indicated that normalized blood pressure following continuous norepinephrine administration during anesthesia and open abdominal surgery was associated with an unchanged afterload during that period.

IL-6 levels substantially increased immediately after surgery and did not normalize up to the third postop day. The perioperative pattern indicated that IL-6 is not highly associated with either micro-arterial or extravasation in our opinion. ICAM-1 peaks at the first postoperative day and remains elevated up to the third postoperative day, indicating an association to extravasation. Similarly, Klaschik et al. showed that for the same type of surgery, ICAM-1 did not change from preoperatively to the values at the mourning of postoperative day 1 [27] despite longer durations of surgery and higher fluid administration, further supporting our results.

An interfering factor with the results in this study is the utilized artificial intravenous infusion solutions; as especially for starches, it was shown that it can trigger immunological and endothelial reactions and was associated with renal and coagulatory dysfunction [28, 29]. In our opinion, all these negative side effects should trigger an increase in extravasation that we did not see in this study, but we cannot exclude it. However, we cannot exclude any relevant interactions of the infusion solutions, especially the starches, with the endothelium impacting these results.

Clinically, the findings in this study indicate that administration of high amounts of intravenous fluids cannot be justified clinically by the aspect of capillary leakage. On the contrary, in our opinion, it underscores the need to handle fluid administration rationally or even better within a goal-directed hemodynamic algorithm based on advanced monitoring if patients demonstrate during surgery a high demand of intravenous fluids or an unclear volume status. This clinical finding supports the consensus recommendation from the enhanced recovery partnership program by the Department of Health in England [30].

### Limitations of the study

This is a pilot study on vascular evaluation during the perioperative course of multivisceral debulking surgery. However, the number of patients must be considered low, and studies with a higher number of patients and studies in other surgical fields will be required to confirm the results and prove their generalizability. In addition, it must be stated that the measurements were performed on the arms and legs, being a surrogate parameter of other organ vessel beds. Especially for the splanchnic bed, we cannot exclude that extravasation might be of relevance. However, in the previously published paper regarding the methodology, we found a high association to clinical edema as a sign of clinical relevance. It is further important to state that fluid balance is a complex clinical factor depending on the management of fluid administration, extravasation, vascular tone, and characteristics of lymphatic drainage. Here, we only investigated extravasation without characterizing the other aspects.

As potential limitations of VCP, it must be stated that any movement might impact the validity of the measurements. Consequently, the measurement site of the forearm had to be completely exposed and shielded, especially to the surgical area. In addition, during measurements, the site had to be closely observed by the study team. However, as this is a fully automated methodology, VCP validated its results and only offered results after plausibility checks. A further limitation was the noise of the compressor for the rapid cuff inflation.

## Conclusion

The extravasation did not change throughout surgery despite a substantial positive fluid balance administered within a goal-directed hemodynamic treatment algorithm indicating that they were not associated with each other. However, the advanced analysis of venous function indicated an affected venous outflow of the congested blood, raising the issue of venous dilation as a probable reason for fluid demand. The micro-arterial function deteriorated throughout surgery compared with the preoperative value but normalized quickly after surgery.

## Supplementary Information

Supplementary material 1. Diagram 1: Consort flow diagram of the study.

Supplementary material 2. Figure 1: Intraoperative Time Course of hemodynamic parameters within a goal-directed algorithm based on the esophageal Doppler monitoring. Time effects (ANOVA) were conducted to examine the changes in the parameters over time.

Supplementary material 3. Figure 2: Perioperative time course of selected laboratory values. Asterisks indicate * = p<0.05, ** = p<0.01, and *** p<0.001 versus preop values.

## Data Availability

The data sets used and/or analyzed during the current study are available from the corresponding author upon reasonable request.
