# Correction: Perioperative micro‑arterial function and extravasation in cytoreductive ovarian cancer surgery: an observational study

**DOI:** 10.1186/s40635-026-00871-y

**Published:** 2026-02-25

**Authors:** Aarne Feldheiser, Jana‑Jennifer Dathe, Sandra Heinig, Klaus Pietzner, Lutz Kaufner, Oliver Hunsicker, Clarissa von Haefen, Jalid Sehouli, Claudia Spies

**Affiliations:** 1 Department of Anesthesiology and Operative Intensive Care Medicine (CCM, CVK), Charité - Universitätsmedizin Berlin, corporate member of Freie Universität Berlin, Humboldt-Universität zu Berlin, and Berlin Institute of Health, Berlin, Germany; 2 Department of Gynecology With Center for Oncological Surgery, Charité - Universitätsmedizin Berlin, Campus Virchow Klinikum, Berlin, Germany; 3 Department of Anesthesiology, Intensive Care Medicine, and Pain Therapy, University Hospital Knappschaftskrankenhaus Bochum, Ruhr-Universität Bochum, Bochum, Germany

**Correction** **:** **Intensive Care Medicine Experimental (2026) 14:7** 10.1186/s40635-025-00839-4

Following publication of the original article [1], the author’s name “Klaus Pietzner” has been updated.

The original article [1] has been updated.
