# Supplementary material for: Perioperative micro-arterial function and extravasation in cytoreductive ovarian cancer surgery: an observational study
Source: Intensive Care Med Exp. 2026 Jan 25;14:7. doi: 10.1186/s40635-025-00839-4 (PMC12831743; doi:10.1186/s40635-025-00839-4)
Supplement: Supplementary file 1 — Supplementary material 1. Diagram 1: Consort flow diagram of the study. [file 40635_2025_839_MOESM1_ESM.docx]

**Appendix:**

**Title of the manuscript:**

Perioperative micro-arterial function and vascular extravasation in cytoreductive ovarian cancer surgery: an observational study.

**Supplementary Table 1:** Preoperative characteristics of study patients.

| **Parameter** | **Values** |
| --- | --- |
| Age (years) | 58 (51.3; 64.3) |
| Body mass index (kg/m^2^) | 22.4 (20.8; 25.9) |
| Metabolic Equivalent of Task Activity (MET) score | 4.5 (4; 7) |
| Charlson comorbidity index | 6 (6; 6) |
| Preoperative fluid fasting (hours) | 7 (3; 11.5) |
| Preoperative solid fasting (hours) | 23 (18.5; 25) |
| **American Society of Anesthesiology (ASA)** | |
| ASA physical status, n (%) | 2 (6.7) |
| ASA Physical Status II, n (%) | 13 (43.3) |
| ASA Physical Status III, n (%) | 15 (50.0) |
| **Postoperative Nausea and Vomiting (PONV)** | |
| PONV Score = 1, n (%) | 4 (13.3) |
| PONV score = 2, n (%) | 8 (26.7) |
| PONV score = 3, n (%) | 9 (30.0) |
| PONV score = 4, n (%) | 9 (30.0) |
| Ascites present, n (%) | 7 (23.3) |
| **Nycturia present** | |
| No, n (%) | 8 (26.7) |
| 1-2 x per night, n (%) | 18 (60.0) |
| 3-4 x per night, n (%) | 4 (13.3) |
| **Breathing Sounds** |  |
| Vesicular sounds, n (%) | 21 (70.0) |
| Rattling sounds, n (%) | 4 (13.3) |
| Wheezing, n (%) | 1 ( 3.3) |
| **Edema present** |  |
| No, n (%) | 20 (66.7) |
| Moderate (n (%) | 7 (23.3) |
| Anasarca, n (%) | 1 ( 3.3) |

Data are presented as median [Q25, Q75] or n (%) patients, as appropriate.

**Supplementary Table 2:** Data during hospital stay and postoperative characteristics.

| **Parameter** | **Values** |
| --- | --- |
| Time of surgery (min) | 210 (137; 260) |
| Length of stay in post-anesthesia or high dependency care unit (hours) | 20 (4; 44) |
| Hospital length of stay (LOS) (days) | 14 (11; 16.7) |
| Red-packed cells transfused during LOS | 2 (0; 4.8) |
| Fresh-frozen plasma transfused during LOS | 3.5 (0; 12) |
| Time until the first defecation (days) | 5 (2.3; 6) |
| Time from the first oral intake of liquids (days) | 0 (0; 1) |
| Time until the first oral intake of soup/yoghurt (days) | 1.5 (1; 3) |
| Time until the first oral intake of solid food (days) | 3 (2; 6) |
| Intravenous infusions (days) | 6 (5; 6) |
| Removal of last drainage (days) | 6 (4.8; 6) |
| Removal of peridural catheter (days) | 6 (6; 6) |
| Richmond-Agitation-Sedation-Score postoperative day 1 morning (points) | 0 (0; 0) |
| Sequential Organ Failure Assessment (SOFA) one hour after surgery | 4.5 (0.8; 6.3) |
| Sequential Organ Failure Assessment (SOFA) at 4 hours after surgery | 3 (0; 4.5) |
| Sequential Organ Failure Assessment (SOFA) on postoperative day 1 | 1 (0; 3) |
| Acute Kidney Injury stages according to KDIGO |  |
| Stage 1, n (%) | 5 (16.6) |
| Stage 2, n (%) | 3 (10.0) |
| Physiologic and Operative Severity Score for the enumeration of Mortality and Morbidity (POSSUM) score | 51 (45.3; 55.8) |
| Postoperative ventilator therapy, n (%) | 6 (20.0) |

Data are presented as median [Q25, Q75] or n (%) patients, as appropriate.

**Supplementary Table 3:** Haemodynamic data at the start and end of surgery

|  | **First measurement** | **Last measurement** | **p-value, first vs. last** |
| --- | --- | --- | --- |
| Stroke Volume (ml) | 89.5 (70.3; 110.3) | 88 (75.5; 113.8) | 0.3819 |
| Stroke Volume Index (ml*m^-2^) | 52.3 (39.4; 62.9) | 54.8 (41.1; 67.5) | 0.4344 |
| Corrected Flow Time (ms) | 365 (340; 402) | 379 (350; 410) | 0.4344 |
| Heart Rate (1/min) | 68.5 (60.3; 76) | 70.5 (61; 79) | 0.6264 |
| Systolic arterial pressure (mmHg) | 106 (98; 125.5) | 116 (106.3; 124.8) | 0.217 |
| Diastolic arterial pressure (mmHg) | 57.5 (53.3; 66.8) | 59.8 (54; 68.5) | 0.6082 |
| Mean arterial pressure (mmHg) | 76 (69; 87) | 80.5 (73; 88.8) | 0.3439 |
| Central vein pressure (mmHg) | 8.5 (6; 12.8) | 8 (6.25; 13.5) | 0.7454 |
| Cardiac output (liter/min) | 6.1 (4.8; 7.3) | 6.4 (4.9; 8.2) | 0.0898 |
| Cardiac index (liter*min^-1^*m^-2^) | 3.6 (2.6; 4.4) | 3.8 (3; 4.7) | 0.0744 |
| Systemic vascular resistance (dyn*s*cm^-5^) | 1042 (840; 1264) | 837 (727; 1245) | 0.1899 |
| Systemic vascular resistance index (dyn*s*cm^-5^*m^-2^) | 1737 (1287; 2346) | 1407 (1296; 1883) | 0.1499 |
| Temperature (°C) | 35.9 (35.6; 36.3) | 35.95 (35.4; 36.3) | 0.9712 |
| Administration of norepinephrine (µg/min/kg BW) | 0 (0; 0) | 0.085 (0.012; 0.1) | <0.0001 |

Data are presented as median [Q25, Q75]. P-values were calculated using the exact Wilcoxon test. Abbreviations: BW, body weight.

**Supplementary Table 4:** Perioperative time course of interleukin-6 (IL-6) and intercellular adhesion molecule 1 (ICAM-1) plasma values.

|  | **IL-6 (pg*ml^-1^)** | **ICAM 1 (pg*ml^-1^*1000)** |
| --- | --- | --- |
| Pre | 11.1 (3.3; 16.6) | 9.9 (4.0; 14.2) |
| PACU T1 | 255.4 (77.2; 848.6) *** | 6.7 (4.0; 12.1) |
| PACU T2 | 148.5 (40.6; 450.6) *** | 8.2 (5.0; 12.9) ^#^ |
| POD1 | 60.6 (28.4; 159.9) *** ^###^ | 11.0 (5.4; 18.6) * ^###^ |
| POD3 | 18.3 (9.2; 34.8) * ^###^ | 11.0 (4.8; 17.2) ^#^ |

Asterics indicates (*) p<0.05, (***) p<0.001 versus preop values. Sharps represents (#) p<0.05 and (###) p<0.001 versus the values at PACU T1.

**Supplementary diagram 1:** Consort flow diagram of the study.
