# Supplementary figures and images for: Perioperative micro-arterial function and extravasation in cytoreductive ovarian cancer surgery: an observational study
Source: Intensive Care Med Exp. 2026 Jan 25;14:7. doi: 10.1186/s40635-025-00839-4 (PMC12831743; doi:10.1186/s40635-025-00839-4)

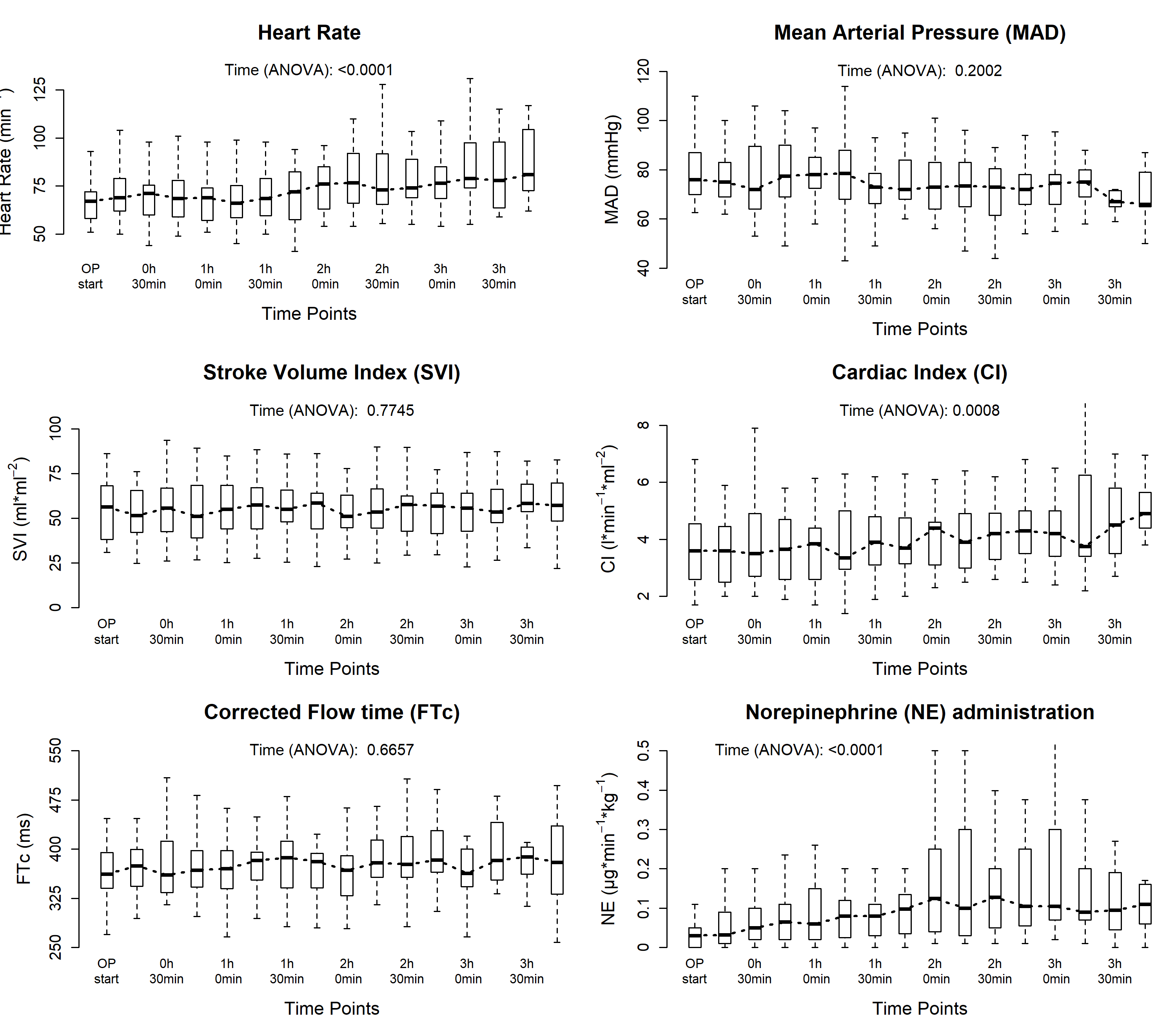

Supplement: Supplementary file 2 — Supplementary material 2. Figure 1: Intraoperative Time Course of hemodynamic parameters within a goal-directed algorithm based on the esophageal Doppler monitoring. Time effects (ANOVA) were conducted to examine the changes in the parameters over time. [file 40635_2025_839_MOESM2_ESM.tiff]
